# Role of fibroblasts in wound healing and tissue remodeling on Earth and in space

**DOI:** 10.3389/fbioe.2022.958381

**Published:** 2022-10-04

**Authors:** Francesca Cialdai, Chiara Risaliti, Monica Monici

**Affiliations:** ASAcampus Joint Laboratory, ASA Res Div, Department of Experimental and Clinical Biomedical Sciences “Mario Serio,” University of Florence, Florence, Italy

**Keywords:** fibroblasts, wound healing, tissue regeneration, microgravity (μg), spaceflight, chronic ulcers, scarring, wound healing dysfunction

## Abstract

Wound healing (WH) and the role fibroblasts play in the process, as well as healing impairment and fibroblast dysfunction, have been thoroughly reviewed by other authors. We treat these topics briefly, with the only aim of contextualizing the true focus of this review, namely, the microgravity-induced changes in fibroblast functions involved in WH. Microgravity is a condition typical of spaceflight. Studying its possible effects on fibroblasts and WH is useful not only for the safety of astronauts who will face future interplanetary space missions, but also to help improve the management of WH impairment on Earth. The interesting similarity between microgravity-induced alterations of fibroblast behavior and fibroblast dysfunction in WH impairment on Earth is highlighted. The possibility of using microgravity-exposed fibroblasts and WH in space as models of healing impairment on Earth is suggested. The gaps in knowledge on fibroblast functions in WH are analyzed. The contribution that studies on fibroblast behavior in weightlessness can make to fill these gaps and, consequently, improve therapeutic strategies is considered.

## Wound healing and tissue regeneration

**FIGURE 1:**
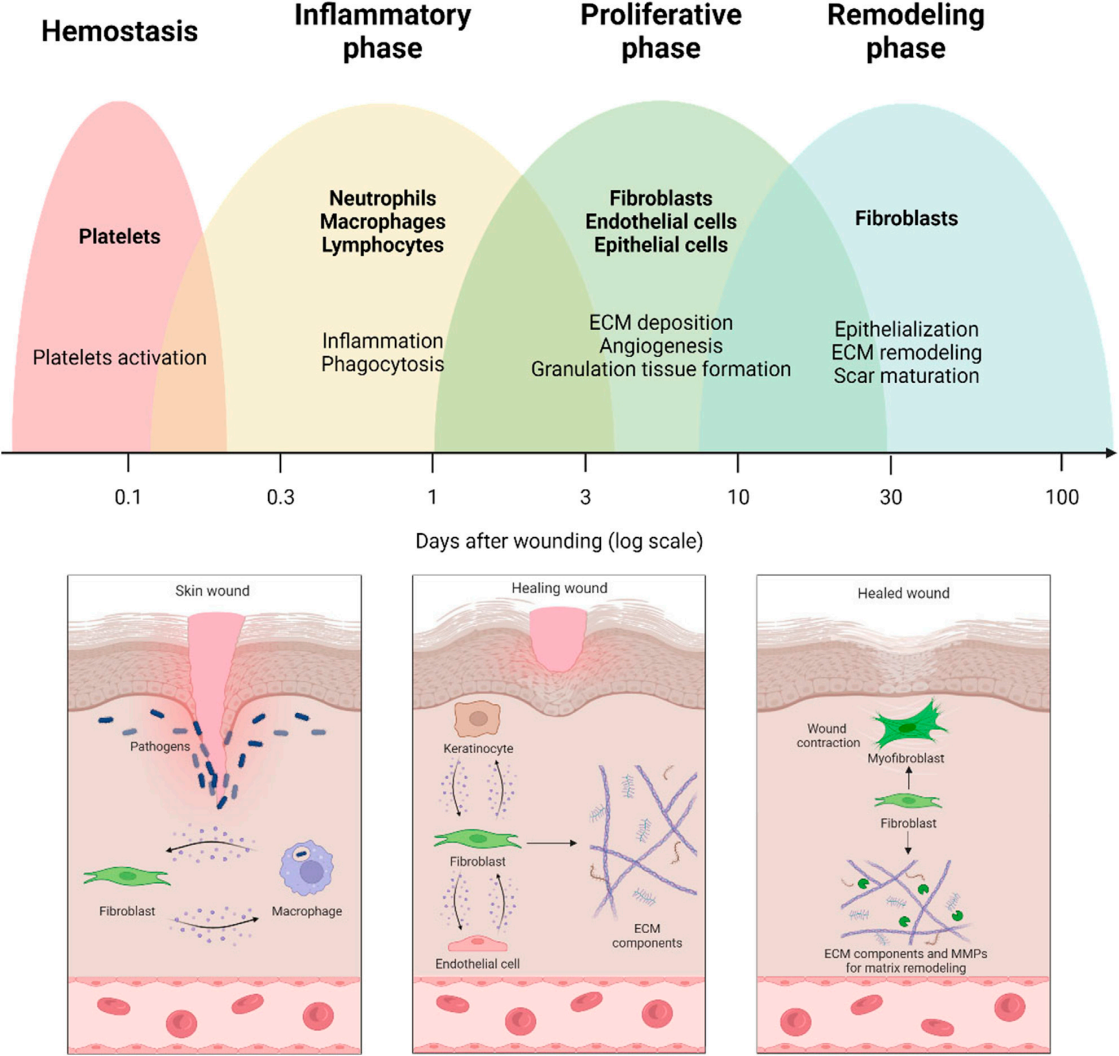
Wound healing process and the involvement of fibroblasts in the different healing phases. The early stages of wound healing are characterized by crosstalk between fibroblasts and immune cells. Thereafter, fibroblasts are responsible for producing extracellular matrix (ECM) components and establishing crosstalk with endothelial cells and keratinocytes. Finally, fibroblasts are involved in remodeling the extracellular matrix by secreting matrix metalloproteinases (MMPs) and matrix components.

The wound healing (WH) process is the response of the organism to an injury. By stopping bleeding and restoring the protective barrier that counteracts the onset of infections and maintains internal homeostasis, WH allows the organism to survive. The healing process is classically described as a sequence of partially overlapped phases, which, after hemostasis, includes inflammation, proliferation, and, finally, remodeling (Figure 1). In reality, each phase includes a series of events or subphases (e.g., early and late inflammation), which are temporally, quantitatively, and qualitatively regulated by a plethora of biochemical and physical factors (Monaco and Lawrence 2003).

The final goal of the healing process is to repair damaged tissues and restore their function. While in invertebrates and non-mammalian vertebrates, the ability to regenerate functional native tissues without scars is quite common, at least in some periods of the life cycle (Arenas Gómez et al., 2020), in adult mammals, with very few exceptions, the progression of healing leads to wound closure with the formation of scar tissue. It is morphologically and functionally different from the native tissue as regards extracellular matrix (ECM) organization (in particular, collagen quality and assembly) and other features: for example, skin wounds heal with scars lacking hairs and glands (Thulabandu et al., 2017). Therefore, the repair is considered successful when it ends with the formation of a limited amount of scar tissue that restores the integrity of the protective barrier and preserves organ function and tissue integrity (Sawant et al., 2021). However, mammalian embryos (including human ones) can regenerate in the early stages of development, and oral mucosa wounds can heal nearly scarless also in adults (Peake et al., 2014).

Despite numerous in-depth studies, how and why adult mammals have lost the ability to regenerate remain unanswered questions. The most accredited hypothesis suggests that, in the course of evolution, given the complexity reached by the mammalian organism, an imperfect but faster healing modality (with scars) has proved to be more advantageous than a perfect but slower regenerative process (without scars) (Cohen, 2007). Intense research is underway to answer this scientific problem because it is a common opinion that bridging this gap of knowledge would allow enormous progress to be made in the management of healing dysfunctions, which heavily affect patient’s quality of life and drain the health system of an enormous amount of resources (Desjardins-Park et al., 2018). A better understanding of the mechanisms for switching from imperfect healing into full regeneration could also be extremely helpful in space medicine from the perspective of future deep space exploration missions.

In case the normal progression of the healing mechanisms fails, chronic conditions arise that result in non-healing ulcers or fibrotic scars. Of the latter, the most common example is skin hypertrophic and keloid scarring. However, internal organs show analogous scarring, in which lesions or pathological conditions induce fibrosis that may or may not resolve over time (Darby and Hewitson, 2007).

Many factors affect tissue repair and its complications: age, gender (due to the different hormone profiles), overweight, systemic diseases (e.g., diabetes), suturing materials and techniques, wound contamination, mechanical factors, emergency care and wound care, and non-physiological environment. Some of these factors cannot be modified, such as age, overweight, and systemic diseases. In contrast, others, such as suturing techniques and wound care, can be managed to facilitate the proper evolution of the healing process. The most common features of healing dysfunctions are persistent inflammation, persistent stromal activation, altered myofibroblast function, uneven areas in the wound bed (i.e., wound areas that are in different healing phases at the same time) with an uncoordinated transition from one phase to another (Darby and Hewitson, 2007).

Despite the progress made in the last 20 years in understanding the origins of healing dysfunctions, their causes are far from clear. Consequently, current therapeutic strategies are poorly effective (DesJardins-Park et al., 2018; Zou et al., 2021).

## The role of fibroblasts in wound healing

Fibroblasts play a crucial role in all three phases of WH. They orchestrate the whole repair process by producing a number of regulatory molecules and crosstalk with the other cell populations involved in the healing mechanisms (Figure 1).

Any injury triggers an inflammatory reaction *via* cytokines deriving from platelet degranulation. Immune cells further increase the level of pro-inflammatory mediators, such as interleukin-1 (IL-1), interleukin-6 (IL-6), interleukin-12 (IL-12), tumor necrosis factor-α (TNF-α), and inducible nitric oxide synthase (iNOS) (Koh and DiPietro, 2011), thus fueling the inflammatory process and stimulating the recruitment and activation of fibroblasts.

During the inflammatory phase, activated fibroblasts engage a crosstalk that strengthens the local immune response and the activation of immune cells in several ways: 1) producing proinflammatory cytokines, such as TNF-α, interferon gamma (IFN-γ), IL-6, and IL-12 (Correa-Gallegos et al., 2021), and releasing a wide range of C–C and C–X–C chemokines, including CXCL1, CX3CL1, and CCL2, to further recruit immune cells to injury sites (Bautista-Hernández et al., 2017); 2) juxtacrine interactions, *via* ICAM1 and CD40 expression, which also activate dendritic cells (Saalbach et al., 2007); 3) remodeling the wound stroma *via* the secretion of matrix metalloproteinases (MMPs) to allow immune cell infiltration; 4) sensing the changing interstitial flow and fluid pressure caused by the inflammatory edema and responding with a modulation of the physical properties of the microenvironment, including rigidity, porosity, elasticity, and viscosity, which make it more immunologically active (Langevin et al., 2013; Correa-Gallegos et al., 2021); 5) migrating collectively from fascia into wounds, thus translocating ECM with embedded immune cells, as recently demonstrated in response to deep injuries (Correa-Gallegos et al., 2019). In summary, fibroblasts can modulate the recruitment of immune cells and regulate their behavior, retention, and survival in damaged tissue. Furthermore, the crosstalk between fibroblasts and macrophages is particularly important in regulating the transition from the inflammatory phase to the subsequent proliferation phase, determining the correct progression of the healing process (Mescher, 2017).

Fibroblast activity becomes even more important during the proliferation phase. After migrating in the provisional fibrin clot, regulated by inflammatory mediators, such as C5a, fibronectin, platelet-derived growth factor (PDGF), fibroblast growth factor (FGF), and transforming growth factor-β (TGF-β) (Thulabandu et al., 2017), fibroblasts proliferate and contribute to angiogenesis and the formation of granulation tissue by secreting pro-angiogenic molecules, including vascular endothelial growth factor (VEGF), FGF, angiopoietin 1 (Ang-1), and thrombospondin (TSP) (Tonnesen et al., 2000; Li et al., 2003). Stimulated by growth factors produced by macrophages and other immune cells, fibroblasts produce MMPs, which degrade the fibrin clot favoring cell migration (Mirastschijski et al., 2010), and ECM molecules, including fibronectin, hyaluronic acid, proteoglycans, and collagen (mostly type III), which replace the fibrin clot with a new provisional matrix supporting keratinocyte migration needed for re-epithelialization (Mirastschijski et al., 2012). Although the proliferative phase can last up to 2 or 3 weeks, starting about 4 days after injury, fibroblasts are the prevailing cell population in the wound, and, stimulated by TGF-β and CXCL8, they begin to differentiate into myofibroblasts (Desmoulière et al., 2005), the protagonists of the remodeling phase.

The final phase of the process can last over a year (Gurtner, 2006) and consists of wound contraction, vascularization and cellularity decline, ECM turnover leading to tissue remodeling, and tensile strength recovery. Myofibroblasts regulate wound contraction and tissue remodeling by combining the ability to synthesize ECM proteins and assume a contractile phenotype (Tomasek et al., 2002; Darby et al., 2014). The fibroblast-myofibroblast transdifferentiation is mainly regulated by TFG-β1 and ECM stiffness. Myofibroblasts assume contractile properties by incorporating α-smooth muscle actin (α-SMA) into stress fibers (Sawant et al., 2021). The contractile activity not only causes wound contraction but also increases ECM stiffness, which in turn induces myofibroblast differentiation and persistence (Grinnell and Petroll, 2010). In the meantime, myofibroblasts remodel ECM through a balanced production of MMPs and ECM proteins, including collagens (Bernardo & Fibbe, 2013). In this phase, collagen III is typically replaced by collagen I, and ECM undergoes a sequential remodeling toward increasing complexity, order, and tensile strength. However, the tensile strength of wounded skin after healing reaches, at best, approximately 80% of that of the unwounded skin (Gurtner and Wong, 2013).

As healing progresses, cellularization decreases through apoptosis (or programmed cell death), which firstly affects the cells of the immune system, then endothelial cells, and finally myofibroblasts. It has been shown that myofibroblast apoptosis occurs when the wound closes and the tissue recovers, at least in part, its tensile strength, suggesting that apoptosis triggers the decrease in vascularization and the evolution from the granulation tissue to scar tissue (Bainbridge, 2013).

## Fibroblast dysfunction in wound healing

Recent research on WH impairment has strongly focused on fibroblasts and their functions involved in the different WH phases. Fibroblast dysfunction can lead to opposite healing problems, namely, fibrosis and healing delay.

Proper timing for resolution of the inflammation is very important for successful healing progression: a persistent macrophage-fibroblast activation state, with excessive production of pro-inflammatory mediators by fibroblasts and further recruitment of immune cells, generates a feed-forward loop leading to altered repair processes from chronic wounds to fibrosis and scarring (Wynn, 2008; Grinnell and Petroll, 2010; Wynn and Ramalingam, 2012). For example, the excessive fibroblast activity, often occurring in large burns and severe injuries, results in hypertrophic scarring and keloid formation (i.e., dysfunctional and disfiguring scar tissue) (Hinz, 2016; Arif et al., 2021). The persistence of myofibroblast activity can also be caused by altered signaling pathways (Leask, 2021), apoptosis failure (Hinz and Lagares, 2020), and excessive mechanical stress, as in the case of high strains at the wound edges (Wong et al., 2011a) or ECM stiffness (Sawant et al., 2021).

Conversely, myofibroblast dysfunctions can also cause delayed wound closure up to chronic ulcers, which fail to heal due to failure in ECM reconstitution. Chronic ulcers are often associated with cardiovascular and/or metabolic diseases, chronic infection, and inflammation (Wan et al., 2021). A study on diabetic mouse fibroblasts, compared to normal ones, found a 75% reduction in migration, altered MMP9 production, and a tremendous decrease in VEGF production (Lerman et al., 2003). Recently, it has been shown that high glucose impairs the proliferation and migration of human gingival fibroblasts (HGFs), explaining the delayed gingival WH in diabetic patients (Buranasin et al., 2018). The outcomes of these studies unequivocally indicate the inability of fibroblasts to promptly migrate to the wound site, properly remodel ECM, and adequately support neoangiogenesis in conditions of high glucose levels (Table 1). Moreover, prolonged inflammation and wound infection can strongly affect ECM production. Persistent inflammation leads to a wound environment characterized by an excess of pro-inflammatory molecules, proteolytic enzymes, and reactive oxygen species (ROS). Both proteolytic enzymes and ROS directly damage ECM molecules, altering the balance between ECM production and degradation, with the prevalence of degradation. In these conditions, the cells involved in the healing process, including fibroblasts, in particular senescent ones, are stimulated to further produce proteolytic enzymes and ROS, generating a vicious circle that hinders the normal evolution of WH. Wound infection further worsens the scenario as it fuels inflammation while its microbial components interfere with cell-ECM interaction (Eming et al., 2007).

**TABLE 1:** Impairment of fibroblast functions involved in wound healing in conditions of high glucose level.

	Normal wound healing	Diabetic wound healing impairment
Migration	↑	↓
VEGF production	↑	↓
Inflammation	↓	↑
ROS	↓	↑
Proteolytic enzymes	↓	↑
ECM degradation	↓	↑

↑ higher; ↓lower; extracellular matrix (ECM); reactive oxygen species (ROS); vascular endothelial growth factor (VEGF).

## Wound healing in unloading conditions

The studies on WH in space, namely, in microgravity (*µg*) conditions, are relatively few. To the best of our knowledge, no studies concerning the effect of spaceflight on WH in humans are available. This knowledge gap is largely motivated by the fact that, in space missions within the low Earth orbit (LEO), the likelihood of surgical emergencies or traumatic events causing serious wounds is very low. In any case, a medical evacuation to Earth is always feasible. However, Space Agencies are aware that future interplanetary missions open completely different scenarios. Realistically, medical emergencies will be more likely and will have to be managed aboard spacecraft and space stations or in space bases. Due to the great distance from Earth, medical evacuation would not be feasible, nor would it be easy to guide crew actions remotely due to communication lag.

Therefore, Space Agencies have for some time been conducting studies on the feasibility of surgery in space and the implementation of dedicated techniques (Panesar and Ashkan, 2018; Pantalone et al., 2021; Rajput, 2021). Already 10 years ago, in a report on the “state of the art” in space surgery, wound management and healing in space was indicated as a critical topic that needed to be studied in depth (Drudi et al., 2012). Hence, Space Agencies have begun to support studies on tissue repair and regeneration mechanisms in the space environment. The ESA-TT on “Tissue Healing in Space: Techniques for Promoting and Monitoring Tissue Repair and Regeneration” and the research projects originating from it are clear examples.

Although there are no studies on WH in humans during space missions, the well-known pathophysiological changes induced by spaceflight could affect the ability of the organism to respond effectively to injuries (Kirkpatrick et al., 2009). For example, the deficient immune function (Crucian et al., 2018), chronic low-grade inflammation (LGI) and metabolic alterations (Strollo et al., 2018), changes in hemorrhage evolution (Kirkpatrick et al., 2009), and skin microbiota (Marvasi et al., 2022) could be contributing causes of healing impairment in space.

Recent studies on models simulating *µg* conditions demonstrated an elevated neutrophil-to-lymphocyte ratio (NLR), confirming previous data collected from astronauts and animal models exposed to real *µg.* Investigating the mechanisms involved, the authors found alterations in redox processes and oxidative stress responses leading to the production of pro-inflammatory mediators (Paul et al., 2020a). These changes could significantly affect the inflammatory phase of WH, compromising its normal evolution and sustaining a deleterious persistence of inflammation. Moreover, the *µg*-induced inhibition of T lymphocyte activation (Hauschild et al., 2014) and dendritic cell maturation (Monici et al., 2007) could interfere with the crosstalk between wound fibroblasts and immune system cells.

Research on the hindlimb unloaded mouse model, alone or in combination with acute simulated galactic cosmic rays or solar particle events irradiation, showed that each condition resulted in distinct circulating immune responses (Paul et al., 2020b). These results demonstrate that, in deep space conditions, immune system dysregulation could be aggravated by stressors other than unloading.

Some *in vivo* and *in vitro* studies demonstrated that µg affects platelets’ number and function, thus increasing the risk of hemorrhages and contributing to delay WH (Locatelli et al., 2021). Moreover, the reduction in circulating blood volume observed in astronauts during spaceflight might impact the body’s ability to withstand blood loss. Finally, changes in skin microbiota during spaceflight might affect WH by predisposing wounds to infections and through interaction with the immune system.

The relatively few studies performed in real and modeled *µg* using animal models did not provide definite results (Riley et al., 1990; Kirchen et al., 1995; Davidson et al., 1999; Campbell et al., 2005; Midura et al., 2006; Delp, 2008; Heinemeier et al., 2009), but most of them showed that the healing of wounds and fractures is delayed and impaired in weightlessness, and alterations have been documented in the three different healing phases (Delp, 2008). Studies performed on rats revealed bone healing impairment, with reduced callus formation and angiogenesis (Kirchen et al., 1995; Midura et al., 2006). Defective microvasculature was found in injured muscles, suggesting impaired neoangiogenesis and delayed muscle repair (Heinemeier et al., 2009). Reduced growth factor responses and ECM deposition in tendons and ligaments, resulting in decreased strength, have been hypothesized to jeopardize repair mechanisms in connective tissues (Provenzano et al., 2003; Martinez et al., 2007). Recent studies, carried out in simulated *µg* using an *in vivo* WH model in *Hirudo medicinalis*, showed delayed healing and alterations in the newly formed connective and epithelial tissues, which appeared less organized than *1xg* controls and showed a lower collagen fiber density. The addition of platelet-rich plasma (PRP) to the culture medium during exposure of injured leeches to *µg* prevented, at least in part, both healing delay and alterations in tissue structure. These results suggest considering PRP among the possible countermeasures for *µg*-induced WH impairment (Cialdai et al., 2020).

Although *in vitro* experiments specifically focused on WH in weightlessness are relatively few, the literature offers several studies concerning the effects of *µg* on the behavior of cell types that, together with fibroblasts, play key roles in WH. About immune cells, it is well known that lymphocyte activation is impaired in *µg,* whereas granulocytes seem to be overactivated, although their behavior in weightlessness has been little investigated (ElGindi et al., 2021). Studies concerning macrophages, recently reviewed by Ludtka et al. (2021), showed *µg*-induced alterations in cell metabolism, signal transduction, proliferation, cytokine secretion, differentiation, cytoskeletal structure and morphology, migration, gene expression, and inflammatory response (Thiel et al., 2019; Vogel et al., 2019; Shi et al., 2021). Furthermore, endothelial cells, responsible for angiogenesis, are very sensitive to mechanical and gravitational stresses. It has been demonstrated that, when exposed to *µg*, endothelial cells show cytoskeleton remodeling, mitophagy, changes in proliferation, apoptosis, adhesion molecules, migration in response to chemoattractants, production of ECM components, and vasoactive and inflammatory mediators (Morbidelli et al., 2005; Maier et al., 2015; Kruger et al., 2019; Locatelli et al., 2020). Research on keratinocytes demonstrated that exposure to *µg* initially induces changes in gene expression profile, rearrangement of cytoskeleton, and cell–cell and cell–matrix interactions, leading to enhanced epithelial–mesenchymal transition. Later, these changes are reversed and followed by adaptive modifications through which the cells miss the acquired mesenchymal phenotype (Clement et al., 2008; Ranieri et al., 2017; Ricci et al., 2021). Recent studies focused on mechanisms involved in WH have shown that fibroblast behavior is strongly affected by *µg.* The results of these studies will be described in detail in a dedicated paragraph.

In summary, *in vitro* studies show that lymphocytes, granulocytes, macrophages, fibroblasts, endothelial cells, and keratinocytes are sensitive to *µg*. It induces significant alterations in the cell functions involved in WH, such as migration, proliferation, differentiation, apoptosis, production of cytokines, growth factors, and ECM molecules. In agreement with what has been observed *in vitro*, research on animal models shows delayed healing, angiogenesis impairment, ECM disorganization, and, consequently, morphological alterations in tissues. These results and the possibility that pathophysiological changes induced in the human organism by spaceflight (in particular, immune dysfunction, increased low-grade inflammation, and metabolic alterations) interfere with repair mechanisms suggest that WH could be delayed and impaired in space. Therefore, studies on WH in spaceflight conditions and the development of strategies to manage wounds and burns on board spacecraft or space stations are needed in view of future interplanetary missions.

## Alteration of fibroblast function in space and possible consequences on wound healing

Literature offers a still limited number of studies on the behavior of fibroblasts in *µg*, mostly published in the last 2 decades (Table 2). The early studies aimed to understand whether fibroblasts, the most representative cells of connective tissue proper, were sensitive to *µg* conditions, as had already been ascertained for bone and immune system cells, belonging to specialized connective tissues. Since it was an almost entirely unknown field, the objective of these early studies was to obtain a general overview of the alterations fibroblasts underwent in *µg*.

**TABLE 2:** Summary of selected articles addressing research on fibroblast behavior under real and simulated microgravity.

Experimental model	Device and exposure duration	Findings in microgravity (μg)	References
Human fibroblasts	3-D clinostat (24 h)	Upregulation of XRCC1, TNF, ICAM-1. Downregulation ofERB-B2, p21	Arase et al. (2002)
Human fibroblasts (WI38)	Space Shuttle during the STS-93 mission (4 d and 23 h)	Upregulation of genes from TNF superfamily, TNF-inducible genes, TNF-α, IL-1 receptor antagonist and downregulation ofIL-15 receptor α chain	Semov et al. (2002)
Human fibroblasts (WI38) quiescent cells	Space Shuttle during the STS-93 mission (5d)	Changes in gene expression associated with oxidative stress and DNA repair pathways. Downregulation of genes involved in energy metabolism	Liu and Wang (2008)
Normal human foreskin fibroblasts (AG1522) confluent culture	International Space Station (3d and 14d)	At 3d (slowly proliferating cells): activation of growth-related pathways and down-regulation of the Let-7 miRNA family	Zhang et al. (2016)
		At 14d (not proliferating cells): minimal changes in gene and miRNA expression profile	
Mouse embryonic fibroblasts (MEF) and Mdc-1-deleted MEF	RCCS (from 1d to 5d)	Increased SMG-induced DNA double strand breaks (DSBs) in Mdc-1-deleted cells but not in wild type cells. Partial adaptation (reduction of DNA damage) at 5d. ROS only partially responsible for SMG-induced DNA damage	Li et al. (2015)
Limbal fibroblasts (LFs)	RCCS (3d)	Lower proliferation rates and higher proportion of cells expressing CD90, CD105 and SSEEA44	Pao et al. (2017)
Human dermal fibroblasts	Space lab D2-mission 1993 (4,7,10 and 20 h)	Increased collagen synthesis. No effect on relative proportions of synthesized collagens I, III, and V	Seitzer et al. (1995)
Juvenile normal human dermal fibroblasts (NHDF)	RPM (3 d)	Changes in cytoskeleton organization and focal adhesion molecules. Presence of two phenotypes (part of the cells grew as 3D aggregates (spheroids), while the remaining part continued to grow as a monolayer adhering to the plate. Overexpression and intracellular increase in collagen type IV, in parallel with a decrease in collagen type I. Increase in MMP-1 and MMP-3 expression	Buken et al. (2019)
Cardiac fibroblasts (CF) derived from porcine hearts	RPM (24 h)	Increased apoptosis. Increased synthesis of ECM proteins. VEGF and bFGF can revert these effects	Ulbrich et al. (2010)
Rat dermal fibroblasts	RPM (4h or 24h)	Decreased adhesion. Reduced expression of proteins involved in cell-surface interaction	Loesberg et al. (2007)
Fetal fibroblasts	RPM (3d)	Decreased collagen I expression. Increased expression of fibronectin, actin and membrane integrins, reorganization of actin filaments, redistribution of membrane integrins and dysregulation in cell adhesion properties	Monici et al. (2011)
Fisher 344 rats	Space Shuttle Endeavour (STS-57) (9d)	Decrease in collagen amount. Delayed wound healing	Davidson et al. (1999)
NIH-3T3 murine fibroblasts	RCCS (3d) w or w/o Platelet Rich Plasma	Rearrangement of cytoskeleton, impaired adhesion ability and inhibition of migration. Decreased expression of alpha-SMA and E-CAD	Cialdai et al. (2017)
		PRP is effective in partly restoring fibroblast chemokinetic properties compromised by SMG	
NIH-3T3 murine fibroblasts and leech model	RPM w or w/o Platelet Rich Plasma [fibroblasts (3 d); leech (6 h, 2 d, 4 d)]	Fibroblasts: decrease in migrating ability and alpha-SMA expression	Cialdai et al. (2020)
		Leech: delayed healing and structural alterations in the repair tissue. PRP partially counteract these effects both in vitro and in vivo models	
Human dermal fibroblasts	RPM (24h)	Increased apoptosis and reduced proliferation. Oxidative damage. Compromised migrating properties. Downregulation of fascin, α-SMA, cofilin actin and vinculin. Impaired fibroblast-keratinocyte cross-talk	Fedeli et al. (2022)
Human dermal fibroblasts cultured within collagen matrix	RPM (3d)	Decrease in alpha-SMA and Smad 2/3 expression. Reduction of matrix remodeling	Sapudom et al. (2021a)
Human fibroblasts (1BR-hTERT cells)	3D-clinostat (24h) + radiation (heavy-ion beam and x-ray)	Increased number of chromosome aberrations (CA) respect to radiation alone	Hada et al. (2018)
Human fibroblasts (1BR-hTERT cells)	3D-clinostat (24h) + radiation (heavy-ion beam and x-ray)	Downregulation of cell cycle-suppressing genes, such as p21, and upregulation of genes promoting cell cycle progression	Ikeda et al. (2019)
STO mouse fetal skin fibroblasts	Irradiation with increasing doses of X-ray then RPM (24h)	Decrease in apoptosis (lower level of caspase-3 activity)	Beck et al. (2012)
STO mouse fetal skin fibroblasts	RPM (65h) or ionizing radiation (55mSv) (65h) or RPM + ionizing radiation	Microgravity induced upregulation of oxidative stress responsive genes, such as targets of the nuclear factor-erythroid 2 p45-related factor 2 (Nrf2). Radiation decreased expression of genes involved in cytoskeleton remodeling. The expression of these genes changed in the combined treatment, indicating that the interaction between effects induced by *μg* and radiation is complex to be understood	Beck et al. (2014)

α-SMA, alpha-smooth muscle actin; bFGF, basic fibroblast growth factor; CF, cardiac fibroblasts; CA, chromosome aberrations; CD105, cluster differentiation 105; CD90, cluster differentiation 90; p21, cyclin-dependent kinase inhibitor 1; DSBs, double strand breaks; E-CAD, E-cadherin; ERB-B2, Erb-B2 Receptor Tyrosine Kinase 2; ECM, extracellular matrix; ICAM-1, intercellular adhesion molecule 1; IL-1, interleukin-1; IL-15, interleukin-15; Let-7 miRNA, Let-7 microRNA; LFs, limbal fibroblasts; MMP-1, matrix metalloproteinase 1; MMP-3, matrix metalloproteinase 3; Mdc-1, mediator of DNA, damage checkpoint 1; MEF, mouse embryonic fibroblasts; NIH, national institute health; NHDF, normal human dermal fibroblast; Nrf2, nuclear factor-erythroid 2 p45-related factor 2; RPM, random positioning machine; ROS, reactive oxygen species; RCCS, rotating cell culture system; SIM, sandos inbred mice; STO, thioguanine and ouabain-resistant; SMG, simulated microgravity; STS-93, Space Transportation System 93; SSEA4, stage-specific embryonic antigen-4; TNF, tumor necrosis factor; VEGF, vascular endothelial growth factor; XRCC1, X-ray repair cross-complementing protein 1.

Mainly based on the analysis of genomic profiling, these studies provided a broad picture of *µg*-induced changes affecting multiple aspects of fibroblast behavior, such as growth, signaling, adhesion, transcription, and apoptosis. In detail, a study published by Arase et al. (2002) examined the effect of a 24 h exposure to simulated *µg* (3D-clinorotation) on the gene expression level in human fibroblasts. Among the 588 genes examined, the most significant results were as follows: 1) upregulation of *XRCC1,* a repair-related gene that the authors hypothesized to be involved in the response to *µg*; 2) downregulation of *ERB-B2*, a proto-oncogene not directly involved in gravity-dependent signaling cascade but associated with integrins, which have been shown to be important in the transduction of mechanical stress; and 3) reduced expression of p21, a strong inhibitor of the cell cycle. In the same year, other authors identified 10 genes whose expression levels were altered in human WI-38 fibroblasts after 5 days aboard the Space Shuttle. These genes belong to the TNF or IL-related gene families and are thought to be involved in either the regulation of bone density, connected with the development of spaceflight osteopenia, or the development of the pro-inflammatory status (Semov et al., 2002).

In a subsequent Space Shuttle mission, a similar experiment was carried out by Liu and Wang (2008). In human WI-38 fibroblasts analyzed after 5 days of spaceflight, they found an increase in the expression of transcripts encoding oxidative stress response and DNA repair pathways, which could impact cell apoptosis and senescence. The downregulation of genes involved in energy metabolism was also observed. In contrast, Zhang et al. (2016) reported that exposure to the space environment for 3 or 14 days on board the ISS induced minimal changes in the gene and miRNA expression profile of human fibroblasts (AG1522). It is important to point out that, in this experiment, confluent fibroblast cultures were used. In fact, the purpose of the study was to investigate if and how non-proliferating cells sense *µg*, in contrast to previous studies that focused on the effect of *µg* on cell growth and differentiation. Although minimal, the changes were dependent on the number of days of exposure to *µg* and culture conditions. Although the activation of growth-related pathways and downregulation of the Let-7 miRNA family (developmental regulator) were found after 3 days (slowly proliferating cells), after 14 days (not proliferating cells), the gene and miRNA expression profiles of ground controls and flight samples were indistinguishable.

Li et al. (2015) studied the effect of simulated *µg* (3D-clinostat) on DNA damage using mouse embryonic fibroblasts deleted for *Mdc-1*, an important component of DNA damage response, and wild-type cells. They found that 24 h exposure to *µg* induced significant ROS production and DNA double-strand breaks (DSBs) in Mdc-1-deleted cells but not in wild-type cells. As exposure increased, ROS levels returned to control and DNA lesions gradually decreased, though not to control levels, indicating a partial adaptation of fibroblasts to *µg*. The authors concluded that *µg* increased genomic DNA instability in DNA damage response-deficient cells and that ROS were only partially responsible for *µg-*induced DNA lesions.

More recent research studied the effects induced by simulated *µg* (RCCS) in limbal fibroblasts (Pao et al., 2017). These cells possess mesenchymal stem cell characteristics and multilineage differentiation potential (Katikireddy et al., 2014). After 3 days of exposure to simulated *µg* (RCCS), limbal fibroblasts showed lower proliferation rates and higher proportions of cells expressing MSC markers as CD90, CD105, and SSEEA44. Hence, the authors inferred that the differentiation potential of limbal fibroblasts was enhanced by simulated *µg*, opening interesting perspectives for reconstructive therapy in patients with corneal disease.

These studies on the behavior of fibroblasts in µg, despite being conducted with different types of fibroblasts and in different experimental conditions (true or simulated µg, proliferating or non-proliferating cells), showed alterations concerning signaling pathways related to inflammation, repair, metabolism regulation, and oxidative stress response.

As extensively described in the previous part of this review, fibroblasts play a key role in tissue homeostasis because they can act as transducers of biophysical and biochemical cues. They actively participate in WH and orchestrate all the phases of tissue repair/regeneration through the crosstalk with other cell populations involved in the process. Therefore, some studies have been devoted to understanding whether and how *µg* affects fibroblast functions related to WH.

Seitzer et al. (1995) were the first to evaluate collagen biosynthesis by human dermal fibroblasts exposed to unloading and loading conditions. In detail, qualitative and quantitative data on collagen synthesis under altered gravity conditions were investigated by incubating fibroblasts with [^3^H]-proline during space lab D2-mission in 1993. The authors found that *µg* induced an increase in collagen synthesis compared to *1xg* conditions. On the contrary, hypergravity, obtained by means of a hyperfuge, induced a decrease in collagen synthesis, which depended on the *g* value. The relative proportions of synthesized collagens I, III, and V seemed to remain unaffected under any applied conditions.

In a more recent study, overexpression and intracellular increase in collagen type IV, in parallel with a decrease in collagen type I, was observed in juvenile normal dermal fibroblasts cultured for 72 h in simulated *µg* (RPM). A significant increase in MMP-1 and MMP-3 expression was also found. In addition, changes in cytoskeleton organization, focal adhesion molecules, and growth behavior were found. Interestingly, in RPM-exposed fibroblasts, two phenotypes were observed. Part of the cells grew as 3D aggregates (spheroids). In contrast, the remaining part continued to grow as a monolayer adhering to the plate (Buken et al., 2019), suggesting the presence of two different fibroblast subpopulations. The formation of multicellular spheroids in porcine cardiac fibroblast cultures exposed for 24 h to simulated *µg* (RPM) had previously been reported by Ulbrich et al. (2010). They also observed a significant increase in apoptosis, which reverted by adding VEGF and bFGF.

In a study aimed to understand whether cell morphology, orientation, and adhesion properties are more dependent on *µg* or substrate micro-topographical features, rat dermal fibroblasts cultured in simulated *µg* (RPM) on smooth and microgrooved surfaces, respectively, were compared. The authors found that fibroblasts adjusted their shape and orientation according to micro-topographical features, even if simulated *µg* decreased cell adhesion, as proved by the reduced expression of proteins involved in cell-surface interaction (Loesberg et al., 2007). Further confirmation that *µg* significantly affects the production of ECM molecules and cell-surface interaction was obtained in a study on fibroblasts and endothelial cells, protagonists of tissue remodeling and angiogenesis, respectively. After 72 h exposure to simulated *µg* (RPM), a significant decrease in collagen I expression, coupled with increased fibronectin expression, was found in both cell types. In correlation with the increased fibronectin fibrillogenesis, increased expression of actin and membrane integrins, reorganization of actin filaments, redistribution of membrane integrins, and dysregulation in cell adhesion properties were also observed. Interestingly, this study showed that near-infrared (NIR) emission of a Nd:YAG laser source (high power laser, 1,064 nm wavelength) is able to modulate the formation of ordered arrays of fibronectin fibrils and favor endothelial cell monolayer formation. Therefore, it could be investigated as a countermeasure against *µg*-induced effects on tissue remodeling (Monici et al., 2011).

A significant reduction in collagen amount was also demonstrated in an *in vivo* experiment aimed at investigating, for the first time, cutaneous WH in *µg*. In that study, sponge discs were subcutaneously implanted in the ventral panniculus carnosus of Fisher 344 rats sent in the orbiting Space Shuttle for 10 days. Sponges contained growth factors known to accelerate granulation tissue formation. As mentioned above, the authors observed that the spaceflight environment significantly decreased collagen concentration at the injury site compared to ground controls, regardless of the presence or absence of growth factors. Therefore, the authors hypothesized delayed WH in rats exposed to *µg*, although in this experiment, they could not distinguish the effects of *µg* from those of other space stressors (Davidson et al., 1999).

In a study concerning the effect of *µg* on fibroblast functions involved in WH, NIH-3T3 fibroblasts exposed for 72 h to simulated *µg* (RCCS) showed an important rearrangement of cytoskeleton together with an impaired adhesion ability and inhibition of migration in response to chemoattractants. Consistent with alterations in cytoskeleton and adhesion/migration, a decrease in E-CAD and α-SMA expression was found. As α-SMA is the major marker of fibroblast-myofibroblast transdifferentiation, its downregulation suggests that *µg* could affect the differentiation toward the myofibroblastic phenotype, which is a key event in the healing process. Moreover, a decrease in VEGF expression, which could compromise the crosstalk between fibroblasts and endothelial cells, and an increase in COX-2 expression, indicative of induction of an inflammatory phenotype, were also reported. In the same study, PRP, known to promote WH on Earth, proved to be effective in partly restoring fibroblast chemokinetic properties compromised by exposure to modeled *µg*. This finding strengthens the possible use of PRP as a countermeasure to promote WH on Earth and in *µg* conditions (Cialdai et al., 2017).

PRP effectiveness as a countermeasure was the subject of a second study carried out by the same group*. In vitro* and *in vivo* WH models, developed by performing the scratch assay in NIH-3T3 fibroblasts and sutured wounds in the leech, respectively, were exposed to simulated *µg* (RPM). Consistent with the results of the previous study, the scratch assay showed a significant decrease in migrating ability and α-SMA expression in *µg*-exposed fibroblasts compared to *1xg* controls. In the animal model, delayed healing and structural alterations of the repair tissue were observed. The effectiveness of PRP in partially counteracting the alterations induced by *µg* was confirmed in both models. In the scratch assay, it partially restored the ability of fibroblasts to migrate, whereas in the *in vivo* model, it promoted and speeded up wound closure (Cialdai et al., 2020).

A very recent study, carried out with dermal fibroblasts exposed for 72 h to simulated *µg* (RPM), showed a similar panel of results: simulated *µg* induced downregulation of molecular markers involved in WH, such as α-SMA, and alteration of cytoskeleton components, such as the modification of the actin-vinculin apparatus, compromising cell contractility and mechanotransduction, and leading to inhibition of fibroblast-myofibroblast transdifferentiation, with consequent alteration of the fibroblast-keratinocytes crosstalk. Moreover, the authors found signs of oxidative damage and advanced the hypothesis that the *µg*–induced oxidative stress may trigger the mechanisms producing the observed phenotypic changes (Fedeli et al., 2022). Fibroblast differentiation in *µg* was also investigated in the study of Sapudom et al. (2021a) using collagen-based 3D models. After 72 h of exposure to simulated *µg* (RPM), the authors found a significant reduction in α-SMA and Smad 2/3 expression and reduced matrix remodeling, evaluated through the expression of collagen and other ECM components. Once more, these results confirm impairment in fibroblasts-myofibroblast transdifferentiation and altered ECM production in *µg*, in agreement with previous studies.

**FIGURE 2:**
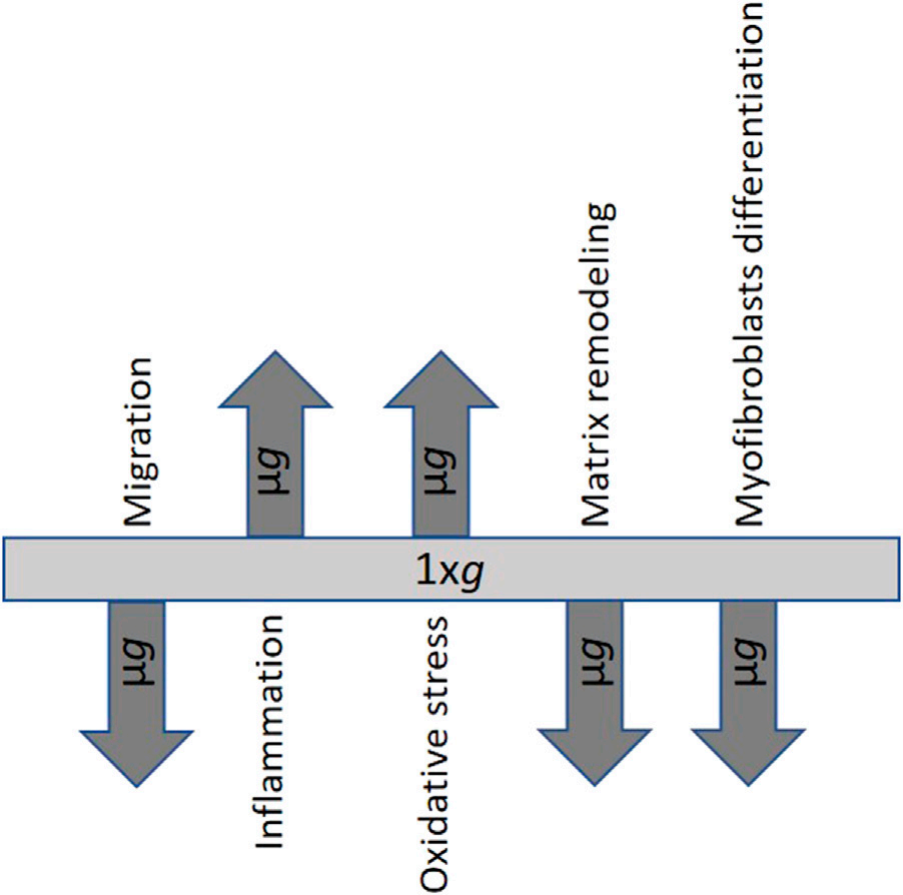
Processes and aspects of fibroblast behavior affected by microgravity. The arrows pointing up and down indicate an increase or decrease following microgravity exposure. Inflammation is intended as an increase in inflammatory markers produced by fibroblasts; oxidative stress is intended as an increase in reactive oxygen species and oxidative processes in fibroblasts.

Although the literature on the behavior of fibroblasts in *µg* conditions is still relatively small and studies have been carried out using different models and exposure conditions, the general overview indicates that unloading conditions strongly affect fibroblast functions, thus jeopardizing the evolution of the WH process. In particular, the results of the most recent studies are very consistent and demonstrate alterations in cytoskeleton and membrane proteins mediating the interaction with the ECM, decreased migration ability, impaired fibroblast-myofibroblast transdifferentiation, alterations in the production of ECM components, signs of oxidative stress, and increase in the levels of inflammation markers (Figure 2). Interestingly, some of these alterations resemble those observed on Earth in the fibroblast dysfunction that characterizes healing impairment. Further studies can help better understand the mechanisms underlying the morphological and functional changes observed in fibroblasts exposed to *µg* conditions. Knowing how and to what extent *µg*-induced fibroblast dysfunction affects tissue healing and regeneration can lay the groundwork for the development of new strategies for counteracting delayed or dysregulated WH in both weightlessness and normal gravity conditions (*1xg*).

Over the years, some studies investigated the combined effect of *µg* and ionizing radiation to better simulate spaceflight conditions in future exploration missions beyond the LEO. In particular, studies were intended to understand whether *µg*, which affects important biological processes, can also modify cell sensitivity to radiation and subsequent repair of radiation damage. By using a 3D clinostat synchronized to a carbon-ion or X-ray irradiation system, research carried out on human fibroblasts simultaneously exposed to simulated *µg* and radiation [heavy-ion beam (0.5 Gy) and X-ray (0.5 and 1.5 Gy)] investigated chromosome aberrations (CA) as a biomarker of cancer risk associated with radiation exposure. In fibroblasts exposed for 24 h to simulated *µg* and radiation, the number of CA was higher than that in cells exposed to radiation alone. The authors inferred that simulated *µg* increased cell sensitivity to radiation and/or reduced cells’ ability to repair radiation damage (Hada et al., 2018). Another study carried out using the same cell model and experimental setup demonstrated that the combined effect of simulated *µg* and radiations (1 Gy of dose both for X-rays and C-ions) induced downregulation of cell cycle-suppressing genes, such as p21, and upregulation of genes promoting cell cycle progression, thereby increasing the risk for events of genomic instability (Ikeda et al., 2019). In addition, Beck et al. (2012) reported that X-rays irradiation (from 0.5 to 4 Gy) followed by 24 h RPM exposure induced in mouse fetal fibroblasts a decrease in apoptosis compared to *1xg* control. This effect could lead to an accumulation of cells with DNA damage and/or mutations.

In a later study, the same authors exposed fetal skin mouse fibroblasts to simulated *µg* (RPM) for 65 h, in combination or not with a low dose of ionizing radiation (55 mSv). The two sample groups were compared to irradiated/non-irradiated *1xg* controls. The authors found that simulated *µg* induced oxidative stress-responsive genes, such as targets of the nuclear factor-erythroid 2 p45-related factor 2 (Nrf2), which is supposed to play a role in cell response to *µg.* Furthermore, the decreased expression of genes involved in cytoskeleton remodeling was observed, possibly mediated by Rho signaling pathway. Radiation alone decreased the expression of genes involved in cytoskeleton remodeling in cell cycle regulation and DNA damage response pathways. Interestingly, *µg* showed a dominant impact on single gene expression, whereas ionizing radiation had a dominant effect on gene sets. Moreover, some genes showing altered expression when exposed to the single treatments (simulated *µg* or irradiation) were not altered in the combined treatment (Beck et al., 2014), indicating that the interaction between the effects induced by *µg* and those induced by ionizing radiation is complex and quite completely unknown.

Although many more studies are needed to shed light on the effects that simultaneous exposure to *µg* and radiation can induce in fibroblasts, the above-mentioned results suggest that the combined effects could further affect fibroblast function, whereas *µg* appears to weaken the response to radiation damage. This result deserves attention because the radiation doses to which crews will be exposed in future missions beyond LEO will be significantly higher.

## Gaps to be filled in research on fibroblast function and wound healing

Although much is known about the role of fibroblast in WH, there are still unanswered questions. The large number of patients suffering from chronic ulcers and fibrosis irrefutably attests that there are gaps in knowledge to be filled in order to implement effective strategies in countering WH impairment.

In comparison to scar models, scar-free WH and skin regeneration models, such as WH in embryos, healing of some wounds in adult oral mucosa, and *Acomys* (the African spiny mouse which can perfectly regenerate skin in wounds as large as 60% of its body surface) show less inflammatory infiltrate, lower CXCL cytokines levels, lower TGF-β1 and TGF-β1/TGF-β3 ratio, higher collagen type III/type I ratio, and higher production of MMPs catabolizing collagen, thus enhancing cell migration and speeding up ECM remodeling, as well as low or absent α-SMA expressing myofibroblasts, which are implicated in scarring (Table 3). The origin of these different characteristics has not yet been understood. However, based on recent research, it has been hypothesized that modifying the behavior of adult fibroblasts and bringing it closer to that of the embryonic ones could be a keystone for tissue regeneration (Thulabandu et al., 2017).

**TABLE 3:** Differences between wound scarring and tissue regeneration.

	Wound scarring	Regeneration
Inflammatory infiltrate	↑	↓
CXCL cytokine level	↑	↓
TGF-β1 level	↑	↓
TGF-β1/ TGF-β3 ratio	↑	↓
Collagen III/Collagen I ratio	↓	↑
MMP catabolizing collagen level	↓	↑
Cell migration	↓	↑
ECM remodeling	↓	↑
Myofibroblasts	↑	↓

↑ higher; ↓lower; C-X-C motif ligand (CXCL); tumor growth factor beta 1 (TGF-β1); tumor growth factor beta 3 (TGF-β3); matrix metalloproteinase (MMP); extracellular matrix (ECM).

Adult human wounds show a spectrum of healing outcomes ranging from nearly scarless healing (close to perfect tissue regeneration) to scarring (including keloids and fibrosis) and delayed/non-healing wounds (including chronic ulcers). Studies on the gene expression profile of fibroblasts derived from these different healing conditions aim to find the corresponding “signatures” and, in particular, identify a signature for enhanced, nearly scarless healing (Peake et al., 2014).

Indeed, for a long time, fibroblasts have been considered a single and fully differentiated cell type. More recent studies demonstrated that fibroblasts are a collection of heterogeneous subpopulations with great morphofunctional plasticity, which can play distinct roles in WH, producing different outcomes of the repair process (Peake et al., 2014; Zou et al., 2021). To date, it is not yet possible to unambiguously identify the different subpopulations of fibroblasts through a combination of surface markers (Jiang and Rinkevich, 2018), and further research is needed to understand the function and behavior of the different subpopulations and distinguish one from the other. It is a common opinion that filling the gaps in knowledge on the specific functions of the various fibroblast subpopulations and how they determine scar quality could allow more effective therapeutic strategies to be developed to prevent scarring and promote tissue regeneration (Jiang and Rinkevich 2020).

Interestingly, a lineage relationship between fibroblasts and adipocytes has been demonstrated during WH. It has been observed that the phenotypic plasticity of fibroblasts toward adipocytes is associated with lower fibrosis (Plikus et al., 2017). Furthermore, in the lung, the reversible myofibroblast-adipocyte transition seems to be associated with reduced fibrosis, whereas the transition of fibroblasts toward a myogenic and contractile phenotype increases fibrosis (El Agha et al., 2017). In the above-mentioned studies, carried out *in vitro* and in animal models, the authors found that the conversion of myofibroblasts to adipocytes (a completely different lineage) is BMP and PPARγ dependent in WH (Plikus et al., 2017) and lung fibrosis (El Agha et al., 2017), respectively. These results pave the way for treatments that, by modulating the plasticity of fibroblasts, direct the healing process toward regeneration rather than scarring.

More than 20 years ago, fibrocytes were described and a leukocyte-fibroblast transition was hypothesized (Bucala et al., 1994). More recent studies indicated that more than half of myofibroblasts in healing wounds derive from myeloid cells and the hypothesis has been advanced of a macrophage-fibroblast transition mediated by extracellular vesicles (EV) released by keratinocytes (Sinha et al., 2018; Guerrero-Juarez et al., 2019). Further research is needed to understand the roots of fibroblast plasticity, if and how it depends on the wound microenvironment, and to what extent it can dictate or shape healing outcomes.

By considering fibroblast plasticity and the variety of subpopulations, an important issue is the crosstalk with the other cell types: it is well known that, in the different healing phases, fibroblasts engage in crosstalk with immune cells, especially macrophages, endothelial cells, and keratinocytes, but it is less clear if, when, and how each different fibroblast subpopulation interacts with the other cell types.

By coinciding with their intense activity at the wound site, fibroblasts undergo transdifferentiation into highly contractile myofibroblasts by developing muscle-like features, including the formation of contractile actin-myosin bundles. It has been widely demonstrated that the phenotype and function of fibroblasts and myofibroblasts are mechanically regulated by matrix stiffness, using a feedback control system integrated with the progress of tissue remodeling (Hinz et al., 2019). However, the interplay between mechanical and biochemical factors in regulating fibroblast subpopulations and its consequences on the healing outcomes are far from clear.

The fibroblast-myofibroblast transdifferentiation is a reversible process controlled by tensile forces (Kollmannsberger et al., 2018), which can sustain the myofibroblast phenotype also in the absence of TGFβ (Jiang and Rinkevich, 2020). The response of fibroblasts to mechanical stress, including that induced by changes in cell–cell and cell–ECM adhesions, involves the Hippo signaling pathway and the nuclear translocation of the Yes-associated protein (YAP) (Dupont et al., 2011). Considerable progress has been made in understanding the molecular mechanisms mediating the fibroblast-myofibroblast transition induced by mechanical stress. However, many aspects remain to be clarified. It would be useful to understand if the various fibroblast subpopulations respond to mechanical stress differently and know if, how, and to what extent the mechanical forces shape fibroblast plasticity.

Studies on 3D fibroblast cultures have shown that wound contraction and closure are driven by fibroblast migration controlled by mechanical forces at the wound edge rather than fibroblast proliferation (Sakar et al., 2016). Both cell–ECM and cell–cell interactions are needed for wound contraction and closure. It has been demonstrated that the actomyosin contraction mechanism and cell–ECM adhesion receptors are crucial for fibroblast mechanosensing and converting mechanical signals of the microenvironment into biological signals (Hinz et al., 2019), which in turn can regulate the fibrotic response. The activation of focal adhesion kinase (FAK) in fibroblasts results in increased scar formation (Wong et al., 2011b), whereas tensile stress reduction results in reduced scarring (Sakar et al., 2016). Furthermore, integrin complexes at cell–ECM focal adhesion sites activate the Hippo pathway and downstream YAP/TAZ, reshaping the actomyosin cytoskeleton as a consequence of changes in ECM (Kim and Gumbiner, 2015). Persistent nuclear expression of YAP/TAZ in fibroblasts supports their mechanoactivation, creating a vicious cycle that increases fibrosis (Liu et al., 2015).

In the wound, tensile forces occurring at fibroblast-fibroblast adhesion sites are as important as those at fibroblast-ECM adhesion sites in modulating wound contraction and scar development. Cadherins, which form the adherens junctions, are integral membrane proteins mediating calcium-dependent cell–cell adhesion (Alimperti and Andreadis, 2015). It has been observed that the expression of cadherin-11 (CDH-11) is higher than normal in the skin of patients with systemic sclerosis or scleroderma, which are autoimmune diseases clinically manifesting as progressive fibrosis of the skin and internal organs (Wu et al., 2014). The growing evidence of the importance of tension forces at the levels of cell–cell and cell–ECM adhesions has suggested various hypotheses on wound contraction mechanisms, and various models of wound contraction have been proposed recently. However, the interplay between tension forces at cell–cell and cell–ECM adhesion sites and their different roles in regulating wound contraction and the development of scars and fibrosis is far from clear (Jiang and Rinkevich 2020).

As *µg* induces alterations in fibroblast function that resemble those observed on Earth in WH dysfunctions, studies in the space environment could represent an interesting model for gaining insights into the molecular and cellular mechanisms leading to healing impairment. Moreover, the space environment (*µg* conditions) offers a unique opportunity to investigate the role of gravity and mechanical forces in WH and mechanotransduction mechanisms in fibroblasts.

While, on ground, the role of fibroblasts in WH has been extensively studied, and the knowledge gaps are quite well defined, in *µg*, the knowledge gaps overwhelm the limited information deriving from the few studies conducted so far. However, this information allows researchers to identify some “main topics” that will certainly need to be studied in depth.

### Fibroblast migration

*In vitro* studies report that *µg* inhibits the ability of fibroblasts to migrate, and this alteration seems to be related to cytoskeletal changes (Cialdai et al., 2017). It has also been reported that *µg* affects the production of ECM components and MMPs (Monici et al., 2011; Buken et al., 2019; Sapudom et al., 2021a). Changes in ECM properties can, in turn, interfere with the migration of fibroblasts and other cell populations involved in WH, such as immune cells, endothelial cells, and keratinocytes. In addition, the production of chemoattractants and the response of fibroblasts to them could change. Therefore, the regulation of fibroblast migration in *µg* needs to be studied in depth.

### Fibroblast regulation by biochemical factors

Fibroblast functions involved in WH are regulated by biochemical factors. The studies conducted so far show that, in *µg*, the levels of growth factors and cytokines produced not only by fibroblasts but also by other cells, such as those of the immune system, can change. Furthermore, in *µg*, fibroblasts seem to respond to biochemical stimuli differently than on Earth (Cialdai et al., 2020). Therefore, the mechanisms of biochemical regulation of fibroblast functions in conditions of altered gravity must be elucidated.

### Fibroblast-ECM dynamics and mechanical cues

The dynamic interaction between fibroblasts and ECM in conditions of altered gravity is quite completely unknown. In *µg, t*he levels of ECM molecules and MMPs produced by fibroblasts change, and ECM properties (relative percentage of components, content of fluids and biochemical factors, mechanical properties, topographical features, *etc*.) are expected to change accordingly. While fibroblasts undergo cytoskeleton reorganization and redistribution of membrane integrins, focal adhesions and the mechanical cues transmitted by ECM to fibroblasts change as well, modulating cell activity. Therefore, the fibroblast-ECM interaction in *µg* might differ significantly from what has been observed on Earth and needs to be investigated. In particular, altered fibroblast-ECM dynamics might strongly affect wound contraction and the remodeling phase of WH.

### Fibroblast crosstalk with other cell populations

It is well known that the crosstalk between fibroblasts and the other cell types involved in WH is extremely important for the proper evolution of the process. Through the production of biochemical factors, cell–cell interactions, and the exchange of materials (e.g., EV), the various cell populations regulate each other and perform their functions concertedly. Particularly important are the interactions of fibroblasts with endothelial cells, keratinocytes, and macrophages, mediating neoangiogenesis, re-epithelialization, the transition from inflammation to proliferation phase, and fibroblast-myofibroblast transdifferentiation (Sapudom et al., 2021b), respectively. In *µg*, these interactions are expected to be altered due to changes in the behavior of the different cell types involved. Moreover, alterations of the microenvironment (e.g., alterations in ECM properties) might interfere with the crosstalk between fibroblasts and the other cell types, including the different immune cell populations.

### Fibroblast plasticity

Last but not least, the fact that, in samples exposed to simulated *µg*, two different fibroblast phenotypes have been observed, one growing as a monolayer adhering to the plate and the other forming spheroids (Buken et al., 2019), show that fibroblast plasticity is manifested even in unloading conditions. As closing the knowledge gap on fibroblast plasticity is considered an essential aspect of progress in the development of new therapeutic strategies, this topic should be investigated even in conditions of altered gravity, and these studies might contribute to better understanding of the subtle differences among various fibroblast subpopulations both in space and on Earth.

## Therapeutic strategies to promote wound healing and tissue regeneration

Delayed wound closure, chronic ulcers, and fibrotic scars are important health and social problems. The wound-care market has been estimated to be over $ 6.5 billion worldwide (Cision, 2011). Unfortunately, it is destined to grow due to the increase in chronic diseases (e.g., diabetes and obesity) and chronic wound patients.

Hence, since ancient times, remedies have been sought to promote successful healing and regeneration of functional tissues. Traditional therapies based on natural origin compounds, such as plant extracts, honey, and products of animal origin, are still used for their low cost, making them the only possible therapy in some parts of the world. Natural compounds show some therapeutic properties, including anti-inflammatory, antimicrobial, and cell-stimulating activities. However, despite advances in extraction and purification methods, the exact mechanisms of action, side effects, and safety of most of these compounds have not been sufficiently studied (Pereira et al., 2013).

Among the pharmacological therapies, antimicrobial agents, including those based on silver, prevent or decrease wound contamination. Corticosteroids and other anti-inflammatory drugs can be useful in attenuating excessive inflammatory response, but they can inhibit fibroblast activity and collagen synthesis, affecting the remodeling phase. In diabetic ulcers, in addition to antidiabetic drugs, estrogens, β-blockers, and ACE inhibitors showed some promising results, acting on microcirculation, angiogenesis, and fibroblast function. Nevertheless, the clinical application of these therapies is often problematic due to the secondary effects of drugs and because the efficacy of some of them is not adequately supported by clinical studies (Spampinato et al., 2020). The possible use of these drugs during space missions will have to be carefully evaluated, as it is known that drug pharmacokinetics and pharmacodynamics can change in the space environment.

Important advances have been made in wound dressing, with the transition from the passive role of traditional protective bandages to the active one of advanced therapeutic dressing systems, such as antimicrobial dressings, anti-inflammatory and analgesic dressings, drug delivery dressings containing pharmacological, biological, or naturally derived agents (Boateng and Catanzano, 2015). Advanced dressings could be useful in the management of serious wounds occurring during space missions. However, the activity of the products they release (antimicrobials, anti-inflammatories, growth factors, *etc*.) must be studied in the space environment, as it could be different from what has been assessed on Earth.

Another category of products that promote WH is blood derivatives such as PRP, platelet-rich fibrin (PRF), leucocyte-rich PRP (LR-PRP), and leucocyte PRF (L-PRF). These products have anabolic effects on fibroblasts, enhance their migration and proliferation, and modulate the expression of genes encoding for ECM and adhesion molecules (Devereaux et al., 2020; Hermida-Nogueira et al., 2020; Akbarzadeh et al., 2021). PRP has already been tested both *in vitro* and in animal model experiments under simulated *µg* conditions. The results show that PRP enhances the activity of fibroblasts, but its effectiveness in *µg* is lower than in normogravity (Cialdai et al., 2020). Therefore, even in the case of blood derivatives, studies are needed to define protocols and dosages for their use in spaceflight conditions.

Physical therapies are used as a single therapy or combined with other treatments to manage wounds and promote healing. One of the most widely used is laser therapy, whose effectiveness is based on anti-inflammatory and antimicrobial actions, as well as on the enhancement of cell energy metabolism. Although there are numerous studies demonstrating the beneficial effects of this therapy, the different types of laser sources and treatment parameters used make it difficult to compare the various studies and develop standardized guidelines and protocols (Suan et al., 2014). Defining the most effective laser sources and treatment protocols could help draw guidelines and achieve unambiguous outcomes. However, a recent study showed that NIR laser radiation effectively controls fibroblast activation induced by cytokine stimulation, thus damping excessive inflammatory response (Genah et al., 2021).

Some difficulties in defining mechanisms of action and treatment parameters affect the use of electromagnetic fields (EMF), whose application in WH has been the subject of numerous studies, with sometimes conflicting results (Pesce et al., 2013; Saliev et al., 2014).

Another therapy showing some effectiveness is Negative Pressure Wound Therapy (NPWT). It can reduce infections, maintain a moist environment, regulate blood flow and exudates, and, by applying pressure, promote wound closure (Orgill and Bayer, 2013).

Hyperbaric oxygen has been applied for the alleged ability to enhance fibroblast proliferation and angiogenesis and improve immune function. However, due to limited effectiveness and the significant side effects, today, this therapy is only considered for ulcers in which a highly hypoxic environment is demonstrated (Han and Ceilley, 2017).

In a space environment, the use of physical therapies could have some advantages. Physical therapies are non-invasive, are generally well tolerated, and have fewer side effects than medications. In addition, they are easy to apply and do not require highly specialized personnel, but only some training. The application of devices for NPWT and advanced laser sources (compact and very safe sources are already marketed and widely applied in clinics) could be feasible, provided that they meet the requirements for use aboard spacecraft or space stations. Furthermore, for physical therapies, studies are needed to evaluate the mechanisms of action in *µg* conditions and develop adequate protocols.

More recent research on advanced therapeutic approaches has been focused on inflammatory mediators, such as TGF-β1; growth factors release in more or less sophisticated ways, from gradual release bandages and scaffolds to gene therapy; modulation of myofibroblast integrins affecting mechanosensing; and regulation of myofibroblast differentiation and epithelial-to-mesenchymal transition (Eming et al., 2007; Schnittert et al., 2018; Sawant et al., 2021).

Despite the promising results in the study phase, the therapies focused on specific targets have not proved to be effective in clinical application. This is probably because WH is a very complex process in which many cell populations and biochemical mediators play their roles according to a precise timeline and often play different roles at different stages of the process. Various tools of regenerative medicine should most likely be used to address the different stages of WH. Furthermore, there are different types of injuries (burns of various degrees, wounds of different depths, etc.) whose healing is strongly influenced by the patient’s condition, particularly in the case of serious diseases, such as diabetes and obesity (Chin et al., 2019). Therefore, the lesson learned is that therapies focused on a single target (or a few targets) have limited efficacy, and, probably, a more holistic approach considering the whole healing process and patient’s conditions is needed.

Skin grafting, that is, skin transplantation, presents some major concerns because it is frequently associated with donor site morbidity, pain, discomfort, and hypertrophic scarring and often requires microsurgery and long healing times (You and Han, 2014). Techniques aimed at developing increasingly advanced skin substitutes utilize hydrogels and 3D bioprinted scaffolds, which can be shaped according to the wound and can incorporate bioactive molecules, such as growth factors, as well as cells, such as fibroblasts, keratinocytes, or stem cells (Murray et al., 2019; Tottoli et al., 2020). These very advanced and promising techniques are mostly in the development phase, and there are no established clinical applications yet. Skin grafting is still the gold standard for severe burns and extensive wounds, but its application in a space environment would be difficult to achieve. The prospects for skin substitutes and regenerative medicine look promising, but these technologies are not yet mature enough. Therefore, Space Agencies started to support research programs aimed at the development of regenerative medicine technologies in a space environment. In summary, to effectively promote healing and prevent scarring, rather than single therapies, there is a need for multifactorial therapeutic strategies (El Ayadi et al., 2020), and further research efforts are needed.

In space, PRP used on a WH model in *Hirudo medicinalis* proved less effective than on the ground (Cialdai et al., 2020). Therefore, the effectiveness of existing therapies will have to be carefully verified in weightlessness, and it will probably be necessary to develop therapeutic strategies that consider the alteration of tissue repair mechanisms induced by *µg*.

## Conclusion

Fibroblasts play a crucial role in WH. After hemostasis, they are involved in all three phases of the healing process: inflammation, proliferation, and remodeling, of which they are the absolute protagonists. Fibroblasts orchestrate the whole WH process through crosstalk with other cell types; production of growth factors, chemokines, MMPs, and ECM components; and the ability to transduce mechanical cues in biological responses and transdifferentiate into myofibroblasts, which are responsible for wound contraction. Therefore, it is not surprising that fibroblast dysfunction is a typical feature of WH impairment, from chronic non-healing ulcers to fibrotic scars, and that most therapeutic strategies aim at regulating and improving fibroblast function. Unfortunately, the many gaps of knowledge that remain regarding fibroblast function in WH negatively affect the development of new and more effective therapeutic strategies. The most important gaps of knowledge concern the plasticity of the fibroblast phenotype and the different fibroblast subpopulations, understanding the roles of the various subpopulations in each WH phase, the different ways fibroblasts use to crosstalk with other cell types, the interplay between mechanical cues, including the mechanical and elastic properties of ECM, and lastly the fibroblast response to them, which can generate a self-sustaining loop.

Studies on fibroblast behavior in weightlessness, a condition typical of the space environment, demonstrated that *µg* strongly affects fibroblast functions, in particular those involved in WH, such as the production of growth factors, pro-inflammatory molecules, MMPs and ECM components, migration/adhesion properties, fibroblast-myofibroblast transdifferentiation, and the crosstalk with other cell types, such as keratinocytes and endothelial cells. These *µg*-induced alterations in fibroblast functions show an amazing similarity with fibroblast dysfunctions observed on Earth in WH disorders ranging from chronic non-healing ulcers to scarring and fibrosis. Therefore, fibroblast cultures in space could represent a useful model to study fibroblast dysfunction on Earth. Moreover, exploring the behavior of fibroblasts in weightlessness is a unique opportunity to gain insights into the regulation of fibroblast functions by mechanical forces and the role of gravity in WH. An interesting effect observed in simulated *µg* is the presence, in the same cell culture, of two phenotypes that differ in the ability to adhere in monolayer or form 3D aggregates. This suggests that fibroblast models in *µg* might help better understand the morphological and functional differences among various subpopulations of fibroblasts.

Progress in the knowledge of tissue repair mechanisms orchestrated by fibroblasts is very important for the development of more effective strategies for wound management and care. Current therapies have limited effectiveness, probably because they target only one or a few steps of the healing process and cannot act on multiple key points. The main goals of current studies are, on the one side, to develop multifactorial therapeutic strategies able to address multiple aspects of the healing process and, on the other side, to identify among fibroblast subpopulations a “signature for nearly scarless healing,” which could pave the way to perfect regeneration. Research on WH and fibroblast models in space, where mechanical stimuli and probably also the ECM properties change, could make a significant contribution to the achievement of these objectives.
